# Adverse Childhood Experiences and Psychological Well-Being in Chinese College Students: Moderated Mediation by Gender and Resilience

**DOI:** 10.3389/fpsyt.2021.710635

**Published:** 2021-08-09

**Authors:** Yafan Chen, Kai Hua, Chienchung Huang, Gaosheng Zhou, Jianfeng Wang

**Affiliations:** ^1^School of Social Work, Rutgers, The State University of New Jersey, New Brunswick, NJ, United States; ^2^Soccer Academy, Wuhan Sports University, Wuhan, China; ^3^Institute of Social Development, Wenzhou University, Wenzhou, China

**Keywords:** adverse childhood experiences, resilience, psychological well-being, gender difference, college students, China

## Abstract

Adverse childhood experiences (ACEs), including child abuse/neglect and household challenges, are a prevalent social issue that impacts individuals' well-being worldwide. Relatively few ACEs studies orient to the presence of psychological wellness, especially in ethnically Chinese populations. Furthermore, less is known about resilience as a mechanism between ACEs and psychological well-being, in addition to the moderating effect of gender. This study examined the relationship between ACEs and psychological well-being among Chinese college students and the potential mediating and moderating effects of resilience and gender, respectively. A total of 1,871 college students studying social science from 12 Chinese colleges completed an anonymous online survey between late September and early October 2020. Multiple-group path analyses were conducted to examine whether the relationships among ACEs, resilience, and psychological well-being differed as a function of gender. Results suggested that gender moderated the relationships studied. For female students, resilience mediated the association between abuse/neglect and psychological well-being, where abuse/neglect was negatively associated with resilience, which in turn had a negative relationship with psychological well-being. For male students, household challenges were negatively related to psychological well-being through reduced resilience. Based on the findings, various ACE-informed initiatives may be essential to prevent and protect individuals from ACEs. We also call for resilience-based interventions to enhance individuals' resilience and thus strengthen their psychological well-being.

## Introduction

Adverse childhood experiences (ACEs) are a prevalent social issue that impacts individuals' well-being worldwide. ACEs usually refer to two broad categories of adverse experiences that occur in the first 18 years of an individual's life: child abuse/neglect and household challenges (1). Child abuse/neglect is defined as physical, sexual, emotional abuse, physical neglect, and emotional neglect toward children. Household challenges refer to five distinct adverse experiences in a household, including parental separation or divorce, domestic violence toward mothers, substance abuse, mental illness, and incarcerated household members (1). Over more than two decades, considerable evidence shows that these early life adversities are pervasive and related to various negative outcomes in a later lifetime (2, 3).

A body of research documents that ACEs are related to a range of mental health outcomes, particularly psychological dysfunction (4–6). In other words, ACE research pays relatively less attention to the presence of psychological wellness, especially in ethnically Chinese populations. Psychological well-being is an important indicator of individuals' positive psychological functioning, and it emphasizes human capacities and the need to flourish (7). Higher levels of psychological well-being are associated with better physical and mental wellness (8, 9) and more desirable job performance (10, 11). Thus, to propel this area of research, this study examines the relationship between ACEs and psychological well-being among Chinese college students and the potential mediating and moderating effects of resilience and gender, respectively.

### ACEs Research in Chinese Populations

The research interest toward ACEs has been grown tremendously over almost two decades (12–15). Using a sample of 2,073 medical college students in Anhui province, Xiao et al. (14) described that more than two-thirds (68.9%) of participants experienced at least one type of ACEs, among which physical neglect (26.9%), physical abuse (26.7%), and mental illness in the household (23.0%) comprised the greatest portions. Subsequently, a number of studies estimated the prevalence of ACEs in China (13, 15–18). Though fluctuating, the rate of ACEs in China is between 35.2–75.0%. In addition to estimating the prevalence of ACEs, researchers have also investigated the potential outcomes of ACEs among Chinese populations. Several studies have shown that ACEs were associated with various health-related outcomes and mental health problems of Chinese individuals (12, 16, 19). For instance, Xiao et al. (14) examined the relationships between ACEs and alcohol-abuse behaviors and found that medical school students with a history of ACEs had a significantly higher risk of alcohol abuse in adulthood compared to those who did not report any ACEs. Analyzing data from a drug rehabilitation center in China, Ding et al. (12) described that ACEs were positively associated with the risk of methamphetamine-associated psychosis.

Most ACE studies, both Chinese and international, examined ACEs using cumulative risk scores and found dose-dependent relationships with studied outcomes [e.g., (20–24)]. Recently, a growing number of studies evaluated the effects of ACEs by category and presented some evidence that different categories of ACEs might have distinct outcomes (17, 25–27). For instance, Anonymous (17) surveyed a college sample from China and found that the abuse/neglect category was related to poorer grit, while household challenges were associated with increased consistency. One study (27) suggested that household challenges and child abuse/neglect had different relationships with various mental health issues. Specifically, child abuse/neglect was significantly related to social phobia, panic disorder, eating disorder, depression, borderline personality disorder, and post-traumatic stress disorder, while household challenges were associated with alcohol dependency and overall psychological distress.

Despite the emergence of ACE studies in China, this area of research still needs further investigation to address some knowledge gaps. First, traditional Chinese culture values family privacy and usually considers child abuse/neglect and domestic violence as family issues that should be kept within the family (28, 29). These values impede the public awareness of the prevalence of ACEs in China and thus underestimate its influences on individuals' psychological well-being subsequently. Thus, the present study is vital to understand the scope of ACEs among Chinese college students and the psychological consequences of the adversity. Second, as reviewed above, the majority of the ACEs studies in China focus on negative psychological outcomes and relatively few concentrated on positive outcomes. To the authors' knowledge, only two studies assessed the relationship between ACEs and positive outcomes among Chinese college students (17, 23). Meanwhile, only Anonymous (17) examined how ACEs related to individuals' psychological outcomes by category in a Chinese context. Moreover, the relationships between ACEs and individuals' outcomes may be complex as various mediators and moderators can interweave. Research should take one step further to explore potential mediators and moderators to understand the nature, scope, and consequences of ACEs on individual well-being more accurately.

### Resilience as a Mediator Between ACEs and Psychological Well-Being

Resilience can help individuals successfully adapt to adversities and promote positive development over time in the face of adversity (30–32). As a multidimensional construction shaped by genetic, neurobiological, epigenetic, psychosocial, and cultural factors (33, 34), resilience has multiple conceptual meanings—(1) an inherent trait, (2) a dynamic process of positive adaptation, and (3) an outcome (30, 34, 35). Though the definitions of resilience vary, there is an agreement that resilience occurs when risk and challenges are present and facilitates individuals' healthy coping to subsequent adversities (36). Individuals with higher levels of resilience are more capable to “bounce back” from detrimental outcomes.

According to Herrman et al. (37), resilience comprises personal factors, biological factors, environmental-systemic factors, and the interaction between personal, biological, and environmental factors. Environmental-systemic factors, specifically, contain the factors from both microenvironmental and macro-systemic levels. Examples of microenvironmental factors include the qualities of interpersonal relationships and parent-child attachment, and the absence of mental illness in parents (37). Child abuse, neglect, and household challenges, three categories of ACEs, fall into the category of microenvironmental factors of resilience and tend to influence individuals' resilience. However, unlikely to promote resilience, ACEs may diminish it—child abuse and neglect could hurt the quality of parent-child relationship/attachment, and different household challenges, such as parental separation/divorce and mental illness in the household, may threaten family stability. Furthermore, trauma theory (38) posits that traumatic experiences, including the majority of ACEs, can impede individuals' mental health and psychological well-being by fixating the feeling of vulnerability and the loss of control of their life and prohibiting their ability to adjust and integrate the traumatic experiences into their personal life. As resilience is a critical indicator of positive adjustment, ACEs as traumatic experiences can dampen it.

Psychological well-being, a crucial indicator of healthy functioning and adaptation, may have a positive relationship with resilience. A massive body of literature has shown that resilience is associated with various psychological outcomes, including but not limited to depression (33, 39), anxiety (40, 41), life satisfaction (42, 43), and both positive and negative affect (44, 45). Particularly, one meta-analysis (46) indicates that resilience has positive correlations with positive indicators of psychological outcomes and negative correlations with negative indicators of psychological outcomes.

Overall, theories and empirical evidence support the mediational role of resilience between ACEs and psychological well-being; however, few studies have investigated mediation pathways. To the authors' best knowledge, only two studies specifically examined the mediational effect of resilience on the relationship between ACEs and psychological outcomes (33, 47). Kelifa et al. (33) study found that resilience partially mediated the relationship between ACEs and college students' depression in Eritrea such that ACEs were associated with reduced resilience, which subsequently exacerbated depression. Another study examined resilience as a mediator between ACEs and post-traumatic growth (PTG) in the U.K. and found that resilience mediated the effects of ACEs on individuals' PTG (47). Unfortunately, we did not find comparable evidence in a Chinese context.

### Relationships Among ACEs, Resilience, and Psychological Well-Being by Gender

The moderation effect of gender on the relationships among ACEs, resilience, and psychological well-being may manifest in several perspectives. First, the prevalence of ACEs could vary across different gender groups (25, 48). For instance, a national survey of 17-year-olds in the U.S. estimated that over one quarter (26.6%) of the female participants had a lifetime history of child sexual abuse, compared to 5.1% of the male counterparts (48). Analyzing 2,235 homeless adults in Canada, Liu et al. (25) found that female participants were likely to report a higher prevalence of 7 out of 10 ACEs items, excluding parental separation/divorce, substance abuse in the household, and incarcerated household members. In another study, Cavanaugh et al. (49) investigated the gender-specific profiles of ACEs among 34,652 U.S adults and demonstrated that female adults had 2–3 times higher probabilities of being in the class of child sexual abuse.

Gender could further modify the relationships among ACEs, resilience, and psychological outcomes. For instance, Haatainen et al. (50) analyzed data from 2,945 adults in Finland regarding ACEs and hopelessness and found that male adults, but not female, tended to report a significantly higher level of hopelessness when there was alcohol abuse in the household. Moreover, Hu et al.'s (46) systematic review reveals that the relationship between resilience and psychological outcomes could be different as a function of gender. However, no known Chinese studies have assessed the potential moderation of gender between ACEs and psychological outcomes.

### Aims and Hypotheses

**Figure 1:**
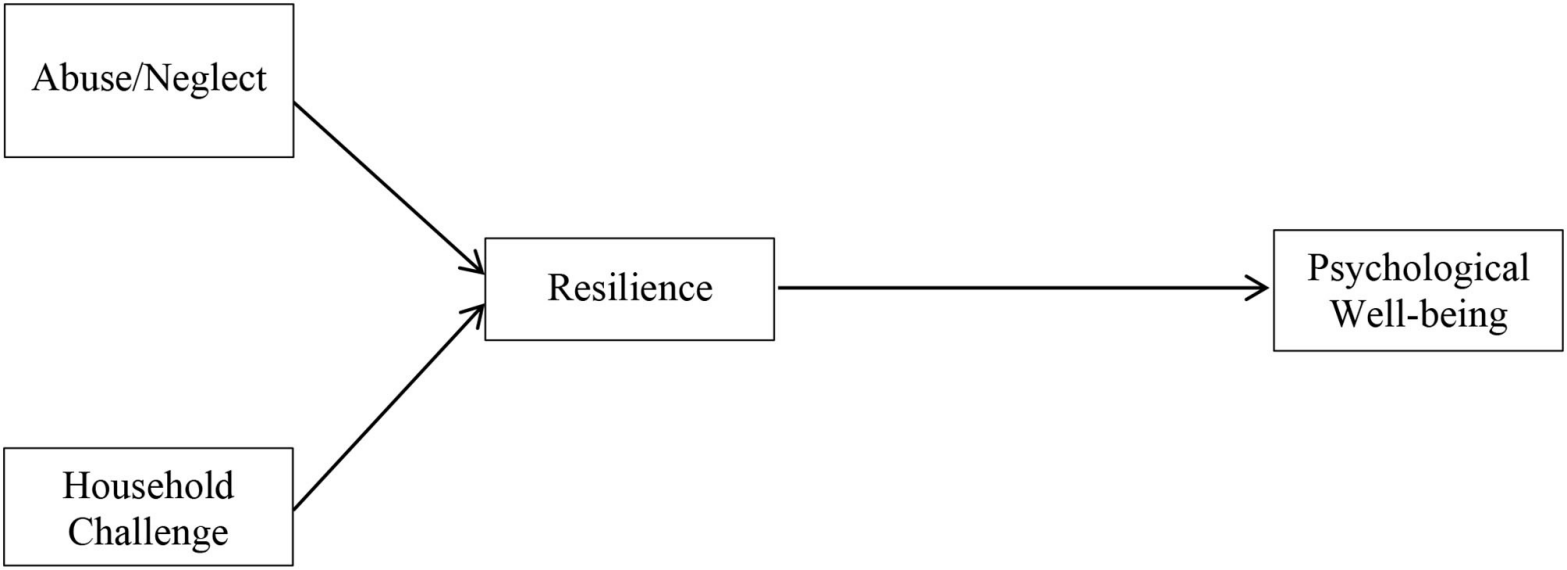
Hypothesized model of ACE dimensions on resilience and psychological well-being.

In summary, a growing number of studies have contributed to the knowledge of ACEs in Chinese populations, including the prevalence and outcomes. However, there are still knowledge gaps regarding whether ACEs are related to psychological well-being and if resilience mediates the relation between ACES and psychological well-being. Further, the moderation of gender on ACEs-related outcomes has yet to be studied in a Chinese context. To fill these knowledge gaps, the current study aims to investigate resilience as a potential mediator of the pathway between ACEs and psychological well-being, using a college sample from China. We hypothesize that resilience mediates the relationship between ACEs and psychological well-being. Specifically, child abuse/neglect and household challenges are negatively associated with resilience, which is positively related to psychological well-being. We further aim to explore whether the relationships among ACEs, resilience, and psychological well-being differ by gender. Given limited evidence of this moderator, this study does not formulate specific hypotheses. The hypothesized model is presented in Figure 1.

## Methods

### Sample and Procedure

The data came from a multi-region online survey taken place in China. In September 2020, we selected 12 universities and invited a total of 2,229 junior and senior students from departments of social science of each university to participate in the survey. To reach a diverse sample, the universities' geographic locations were spread across China, including the north, south, east, west, and middle regions. Students received the initial invitation in late September and two reminders about the survey participation 3 and 7 days later. We provided an incentive of 10 RMB (2 USD) for each participant. By early October 2020, we received 1,881 responses from students. After omitting incomplete and invalid responses, the final sample was 1,871, which yielded a response rate of over 80%. The informed consent process took place before beginning the survey. Students were informed that participation was voluntary and they could choose to terminate the survey at any time. They were also informed that the survey was anonymous and no identifiable information would be collected. The research protocol was approved and overseen by one of the co-authors' institution.

### Measures

#### Dependent Variable

Psychological well-being was measured using the shortened 18-item version of the Psychological Well-being Scale (7). This scale assesses an individual's psychological well-being in six dimensions: self-acceptance, positive relations with others, autonomy, environmental mastery, purpose in life, and personal growth. Some example items include “I like most parts of my personality;” “I live life 1 day at a time and don't really think about the future;” and “I have confidence in my own opinions, even if they are different from the way most other people think.” Students were asked to rate how strongly they agreed or disagreed with the statements on a 7-point Likert scale (from 1 “strongly agree” to 7 “strongly disagree”). Two bilingual social work students translated the scale, and one bilingual social work faculty verified it. We reverse-coded opposite items (i.e., item 1, 2, 3, 8, 9, 11, 12, 13, 17, and 18) and summed up the responses to all items. The scores of the whole scale yielded a range of 18–126, with higher scores representing higher levels of psychological well-being. The Cronbach's alpha was 0.88 in this study.

#### Independent Variable

This study assessed 10 types of Adverse Childhood Experiences (ACEs) during the respondent's first 18 years of life (51). Following previous literature, the assessed ACEs were subdivided into two categories: abuse/neglect (5 items) and household challenges (5 items). The abuse/neglect category encompasses questions about experiences of physical abuse, emotional abuse, sexual abuse, physical neglect, and emotional neglect. Assessed household challenges contained parental separation/divorce, domestic violence toward mother, household substance abuse, mental illness in the household, and incarcerated household members. Example questions include “Did a parent or other adult in the household often: Push, grab, slap, or throw something at you? Or Ever hit you so hard that you had marks or were injured?” “Did you often feel that: No one in your family loved you or thought you were important or special? Or Your family didn't look out for each other, feel close to each other, or support each other?” and “Was your mother or stepmother: Often pushed, grabbed, slapped, or had something thrown at her? Or Sometimes or often kicked, bitten, hit with a fist, or hit with something hard? Or Ever repeatedly hit over at least a few minutes or threatened with a gun or knife?” Respondents answered “yes” or “no” to each question. We assigned one point to each affirmative answer and summed up the items by category. The scores of both categories ranged from 0 to 5. A higher score indicated a greater frequency of the specified category.

#### Mediator

Resilience was measured using Wagnild's (52) 14-item Resilience Scale instrument (RS-14). RS-14 evaluates five characteristics of resilience, including a meaningful and purposeful life, perseverance, equanimity, self-reliance, and existential aloneness (32, 52). Some examples of items include: “I feel proud that I have accomplished things in life;” “I feel that I can handle many things at a time;” “My belief in myself gets me through hard times;” and “When I'm in a difficult situation, I can usually find my way out of it.” Previous studies have demonstrated adequate validity and reliability of RS-14 across racial/ethnic samples (53–55). Meanwhile, the Chinese version also indicates satisfactory reliability among Chinese samples (56, 57). Particularly, RS-14 has been used on Chinese college students and has shown excellent internal consistency (58, 59). Respondents rated each item based on how strongly they identified themselves with the statements in the past 4 weeks from “1” (strongly disagree) to “7” (strongly agree). We summed the graded items to generate a total score of resilience, ranging from 14 to 98. Higher scores represented higher levels of perceived resilience. The Cronbach's alpha of RS-14 in the current study was 0.92.

#### Moderator

Gender was collected from respondents' self-report (female vs. male).

### Analytic Strategies

First, we conducted descriptive and Pearson's correlation analyses to observe the sample characteristics and the correlations among ACEs, resilience, psychological well-being, and gender. Multiple-group path analyses were then conducted to examine the mediation of resilience and moderation of gender. To test the mediating effect of resilience on the path between ACEs and psychological well-being, we used a bootstrapping approach of 5,000 iterations. Path analyses, different from regression analyses, allows the examination of both direct and indirect effects through mediating variables simultaneously. Meanwhile, multiple-group analyses allow one to examine whether the estimated paths within the model are distinct by gender. The multiple-group analyses generated and compared two models: one unconstrained model, in which all estimated paths were allowed to vary across two gender groups, and one constrained model, in which all paths in the model were restricted to be equal in the two groups. We compared the constrained and unconstrained models in chi-square statistics. If the constrained model produced significantly increased chi-square statistics, we concluded that the unconstrained model had a significantly better model-to-data fit, thus paths within the models differed by gender.

The model-to-data fit was evaluated by several fit indices, including Comparative Fit Index (CFI), Normed Fit Index (NFI), and Root Mean Square Error of Approximation (RMSEA). Values of CFI and NFI >0.90 and 0.95, respectively, indicate good model-to-date fit, whereas RMSEA values <0.08 indicate reasonable model-to-date fit. Chi-square statistics are not suitable for this study given it is sensitive to sample size. We used the maximum likelihood (ML) estimation to test the model. The significance of all pathways was assessed by 95% bias-corrected bootstrapped confidence intervals based on 5,000 replications. Analyses were conducted using AMOS 23 (60).

## Results

### Descriptive and Correlation Results

Descriptive statistics and correlations of the main variables are shown in Table 1. The sample reached an average score of 0.43 on abuse/neglect, with a range of 0–5 and a standard deviation (S.D.) of 0.89. The mean for household challenges was 0.26 (S.D. = 0.61). The average score of resilience was 68.64, with a range of 14–98 and an S.D. of 13.42. The sample had a mean of 81.75 on psychological well-being (S.D. = 12.30), ranging from 24 to 121. The correlation analyses indicated that the scores of abuse/neglect and household challenges were positively correlated to each other. However, because the correlation was <0.50, we concluded that no multicollinearity was indicated (61). Table 2 displays the sample characteristics regarding the variables of interest by gender. The distributions of variables of interest did not differ by gender. The sample comprised 66.97% of female and 60.72% of junior students. The average age of the sample was 20.62 years old (S.D. = 0.96). The majority (89.36%) of the sample self-identified as Han ethnicity. Close to 90% (89.04%) of the students' parents were married. Parents' level of education spread across the elementary school to college or above, with college-educated parents occupied the greatest portion (39.82%), followed by junior high school (28.11%), high school or equivalent degrees (25.17%), and elementary school (6.89%). About one-fourth (25.28%) of students' families had utilized social welfare.

**Table 1:** Descriptive statistics and correlations of main variables.

**Variables**	**Mean (S.D.)**	**1**	**2**	**3**	**4**	**5**
1. Abuse/Neglect score [0–5]	0.43 (0.89)	–				
2. Household challenge score [0–5]	0.26 (0.61)	0.43^***^	–			
3. Resilience [14–98]	68.64 (13.42)	−0.17^***^	−0.11^***^	–		
4. Psychological well-being [24–126]	81.75 (12.30)	−0.18^***^	−0.05^*^	0.53^***^	–	
5. Female [%]	66.97	0.03	−0.02	−0.04	0.01	–

*N = 1,871. Standard deviation appears in parentheses*.

* *p < 0.05 and*

*** *p < 0.001*.

**Table 2:** Descriptive statistics for variables by gender.

**Variables**	**Female** **(** ***N*** **= 1,253)**	**Male** **(** ***N*** **= 618)**	
	**Mean (S.D.)**	**Mean (S.D.)**	***F*** **-test**
Abuse/Neglect score	0.45 (0.85)	0.39 (0.96)	1.89
Household challenge score	0.25 (0.55)	0.27 (0.71)	0.50
Resilience	68.29 (12.41)	69.36 (15.25)	2.65
Psychological	81.88 (12.10)	81.49 (12.70)	0.4

### Multiple-Group Path Analyses

For the hypothesized model predicting resilience and psychological well-being, model comparison tests indicated that the constrained model resulted in a significant increase in chi-square statistics compared to the unconstrained model [Δχ^2^ = 47.28, Δ*df* = 3, *p* < 0.001], which was indicative that the unconstrained model had a better model-to-data fit and that the estimated paths varied by gender. The unconstrained model also provided an adequate fit to the data (NFI = 0.97, CFI = 0.97, RMSEA = 0.07). Therefore, we concluded that gender groups were different from one another in terms of relationships between ACEs, resilience, and psychological well-being.

**Figure 2:**
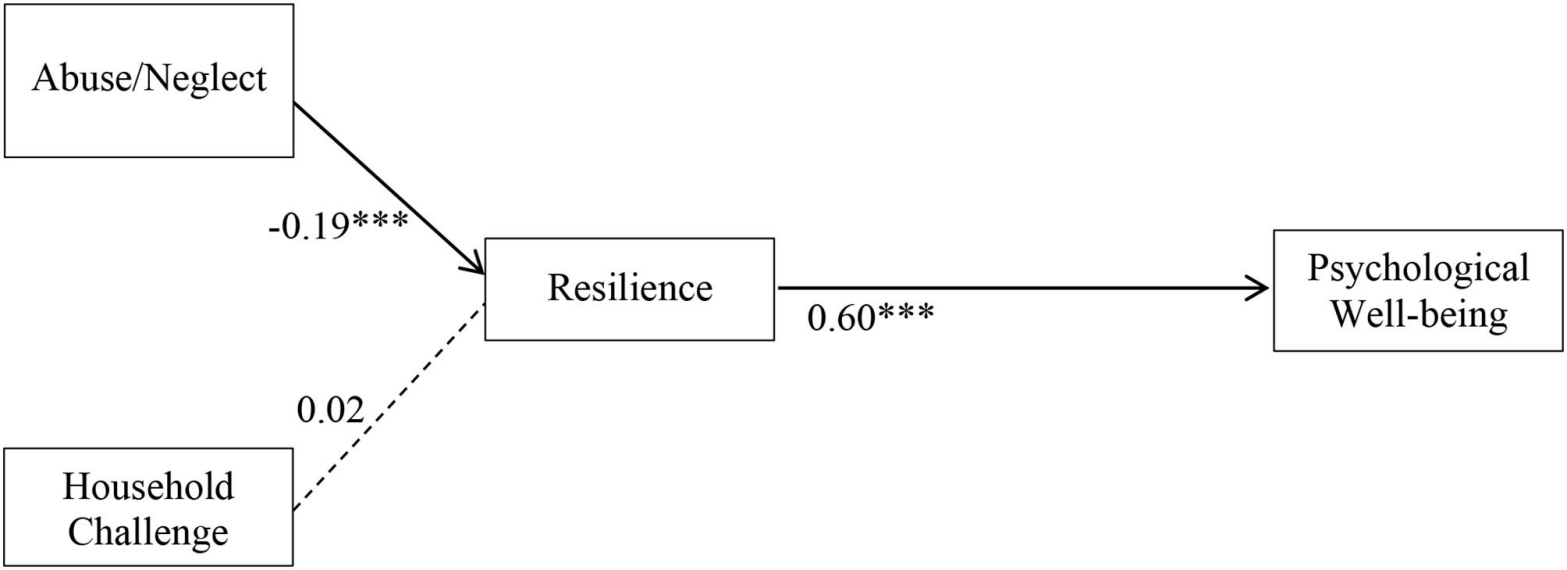
Structural equation modeling of ACE dimensions on resilience and psychological well-being for females. *N* = 1,871. ***p < 0.001. Solid line, significant standardized path coefficient; Dotted line, non-significant standardized path coefficient.

**Figure 3:**
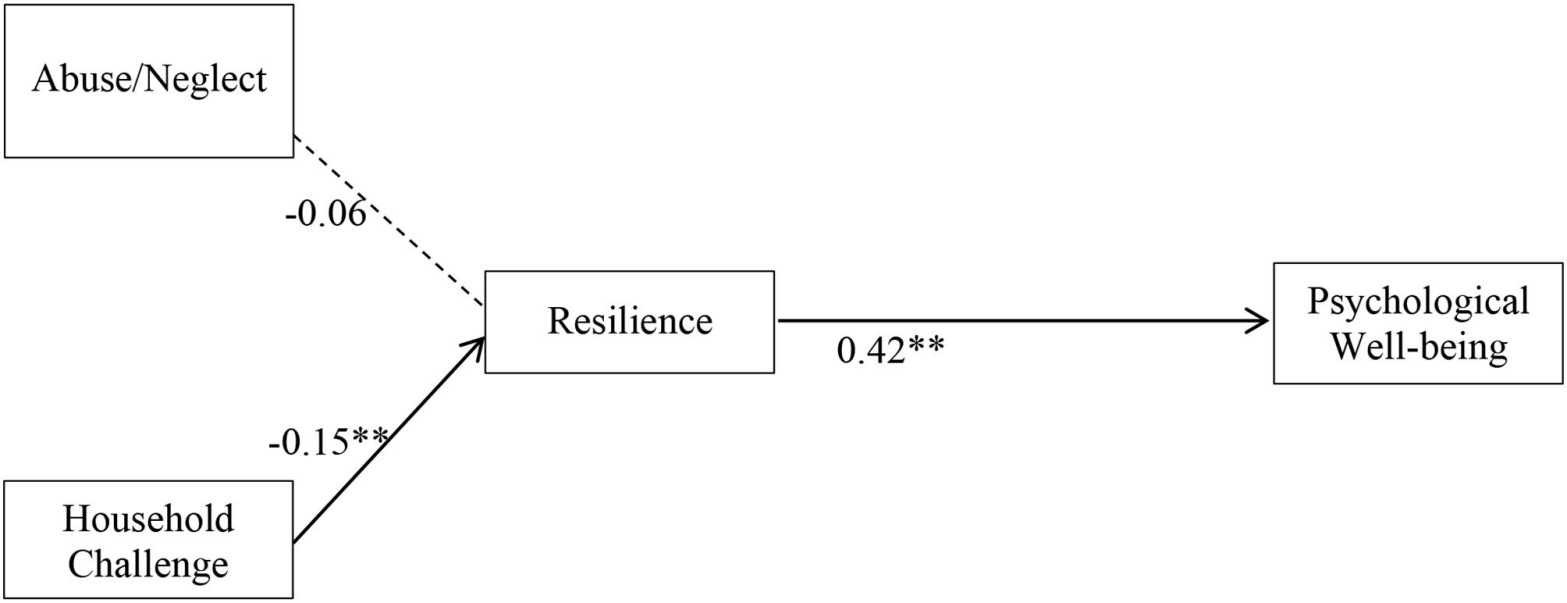
Structural equation modeling of ACE dimensions on resilience and psychological well-being for males. *N* = 1,871. **p < 0.01. Solid line, significant standardized path coefficient; Dotted line, non-significant standardized path coefficient.

Figures 2, 3 display the parameter values and their significance for female and male students, respectively. A bolded line indicates paths with significant standardized coefficients, and a dotted line represents paths with non-significant standardized coefficients. The moderating effect of gender mainly manifested in the relationships between ACEs categories and resilience. For the female group, abuse/neglect had an indirect, negative effect on psychological well-being through resilience such that female students with higher frequencies of child abuse/neglect (β = −0.19, *p* < 0.001) tended to report lower levels of resilience, and those with reduced levels of resilience were likely to have poorer psychological well-being (β = 0.60, *p* < 0.001). Household challenges were not associated with resilience among female students. For male students, household challenges, but not abuse/neglect, were associated with resilience such that male students having more household challenges were prone to have lower levels of resilience (β = −0.15, *p* < 0.01). Similar to the female students, male students' decreased resilience was related to poorer psychological well-being (β = 0.42, *p* < 0.01). Mediation analysis suggested that the indirect effect of abuse/neglect on female students' psychological well-being through resilience was significant (*p* < 0.001), thus, resilience mediated the relationship between abuse/neglect and psychological well-being for female students. Likewise, resilience was a mediator on the pathway between male students' household challenges and psychological well-being (*p* < 0.01).

## Discussion

The findings of this study indicate that the relationships between ACEs, resilience, and psychological well-being differed by gender, and the gender variation was predominately about the relationships among different ACEs categories and resilience. For female students, resilience mediated the relation between abuse/neglect and psychological well-being, while resilience was a mediator on the path between household challenges and psychological well-being for male students. Overall, the findings are aligned with our hypotheses and previous evidence that ACEs are related to psychological well-being and resilience could explain such relationships.

Analyzing the relationships between ACEs and resilience, we found that different categories of ACEs had distinct relationships with resilience in different gender groups. Specifically, child abuse/neglect was negatively associated with female students' resilience, while household challenges were related to lower resilience in male students. Several studies have examined gender differences in ACEs. Some have found the effects of ACEs, particularly household challenges, on certain mental health outcomes to be stronger for male than female participants (25), while others suggested ACEs to be more harmful to females (50). Still, others have found no gender differences in psychological outcomes (62). Given the inconsistent findings of gender differences in ACEs, future investigation into differential influences of ACEs categories on psychological outcomes is warranted.

Consistent with previous research, our investigation reveals that adverse experiences that occurred in childhood could still relate to individuals' resilience and psychological well-being in their emerging adulthood. Additionally, ACEs cause a sizable economic cost in China. In the year 2010, an estimated 0.84% of the gross domestic product (i.e., 50 billion U.S.D) was lost due to child physical abuse. The relative numbers for child emotional and sexual abuse were 0.47 and 0.39% of the gross domestic product in the same year, respectively (63). Although substantial economic costs mentioned above, the public awareness and understanding of ACEs are still yet developed. Thus, increased public awareness is a prerequisite of effective ACEs interventions. Woods-Jaeger et al. (64) interviewed 11 low-income families with young children who had ACEs, regarding parents' suggestions for protecting children from the consequences of ACEs. The parents believed that it was critical to (1) raise awareness about ACEs within the community, (2) establish a supportive community environment, and (3) provide sufficient parenting education and support. Following these suggestions, home-based interventions can be utilized as a preventive tool, that improves caregivers' knowledge of child maltreatment and appropriate parenting behaviors (65, 66). Community-based educational programs may also improve community members' understanding and awareness of ACEs (67). School social workers should provide more comprehensive screening and assessment of the history of ACEs to school-aged children. ACE-informed programs in other venues, such as juvenile justice, mental health facilitates, and healthcare sites can establish a comprehensive response system that addresses ACEs in families and communities (67).

As a mediator of ACEs on psychological well-being, resilience is a vital target of intervention for the population with a history of ACEs. With strengthened resilience, individuals' psychological well-being may be less compromised by ACEs. Several studies have demonstrated the potential of resilience-based interventions on promoting individuals' resilience, which ultimately enhances individuals' well-being [e.g., (68–70)]. Particularly, Chandler et al. (68) examined the feasibility and efficacy of the Empower Resilience Intervention (ERI) among a group of young adults who had a history of ACEs. The study suggested that ERI had the promise to interrupt the detrimental effects of ACEs on illness trajectory. Thus, researchers and practitioners should be responsible for designing and validating effective resilience-based interventions to assist individuals with ACEs.

The prevalence of ACEs in our study only reached the lower bound of the existing range. Zhang et al. (15) collected data from 1,019 Chinese young adults aged between 18 and 21 years old and found that the prevalence of ACEs in this sample was about 75%. In contrast, the sample of our study reported a prevalence of 35.16%. The lower prevalence of ACEs in our study may be explained by the more advantaged socioeconomic status of the sample. For instance, parental level of education is relatively high—about 40% of parents completed college, and one-quarter graduated from high school. Moreover, over 60% of our sample lived in the city, while Zhang et al.'s (15) sample was predominately in rural areas. The urban composition of our sample could lead to a lower prevalence of ACEs as child abuse/neglect and domestic violence are less prevalent in urban areas (71–73). Another potential reason could be participants' social desirability bias (74). Participants could potentially under-report or not report their ACEs concerning the sensitive nature.

There are several other limitations to be considered when interpreting the findings of the current study. First, the completion of the survey was self-report, thus all information gathered may be subject to intentional or unintentional report errors. Particularly, participants, as a group of adults in their early to mid-20s, were asked to recall ACEs in their first 18 years of life. This relatively long reference period may undermine the accuracy of the ACEs responses (75) and ultimately result in under-estimated ACEs prevalence. Second, other unobserved variables could affect psychological well-being but were omitted in the study, such as the quality of peer relationships and ongoing adverse experiences. The absence of the unobserved variables may result in inaccurate results. Third, the data was cross-sectional, thus precluding the examination and establishment of causal relationships between key variables. Future studies should utilize longitudinal research designs to record the change of resilience over time and approximate the variables in a temporal sequence. Fourth, the study was based on college students in social science departments of 12 colleges in China, thus the extent to which the findings could be representative of all Chinese college students is unknown.

## Conclusion

This study investigates the relationships among ACEs, resilience, and psychological well-being, using a group of college students from departments of social science of 12 Chinese colleges. Our investigation suggests the mediating and moderating roles of resilience and gender, respectively. The findings indicate that resilience was positively associated with psychological well-being for both gender groups, but the relationships among ACEs categories and resilience differed by gender. Child abuse/neglect was negatively associated with female students' resilience, while household challenges led to a negative association with male students' resilience. Despite some limitations, this study contributes to the knowledge on the mediation effect of resilience on the association between ACEs and psychological well-being in Chinese college students. Based on the findings, various ACE-informed initiatives may be essential to prevent and protect individuals from ACEs. We also call for resilience-based interventions to enhance individuals' resilience and thus strengthen their psychological well-being.

## Data Availability Statement

The raw data supporting the conclusions of this article will be made available by the authors, without undue reservation.

## Ethics Statement

The studies involving human participants were reviewed and approved by Rutgers University, IRB. Written informed consent for participation was not required for this study in accordance with the national legislation and the institutional requirements.

## Author Contributions

YC, KH, CH, GZ, and JW contributed to conception and design of the study. CH organized the database. YC, KH, and CH performed the statistical analysis. YC wrote the first draft of the manuscript. KH, CH, GZ, and JW wrote sections of the manuscript. All authors contributed to manuscript revision, read, and approved the submitted version.

## Conflict of Interest

The authors declare that the research was conducted in the absence of any commercial or financial relationships that could be construed as a potential conflict of interest.

## Publisher's Note

All claims expressed in this article are solely those of the authors and do not necessarily represent those of their affiliated organizations, or those of the publisher, the editors and the reviewers. Any product that may be evaluated in this article, or claim that may be made by its manufacturer, is not guaranteed or endorsed by the publisher.

## References

[1] Centers for Disease Control and Prevention. Adverse Childhood Experiences (ACEs). (2020). Available online at: https://www.cdc.gov/violenceprevention/aces/index.html?CDC_AA_refVal=https%3A%2F%2Facestudy%2Findex.html (accessed May 19, 2021).

[2] Kessler RC McLaughlin KA Green JG Gruber MJ Sampson NA Zaslavsky AM. Childhood adversities and adult psychopathology in the WHO World Mental Health Surveys. Br J Psychiatry. (2010) 197:378–85. 10.1192/bjp.bp.110.080499 21037215 PMC2966503

[3] Shonkoff JP Garner AS Siegel BS Dobbins MI Earls MF McGuinn L. The lifelong effects of early childhood adversity and toxic stress. Pediatrics. (2012) 129:e232–46. 10.1542/peds.2011-2663 22201156

[4] Burke NJ Hellman JL Scott BG Weems CF Carrion VG. The impact of adverse childhood experiences on an urban pediatric population. Child Abuse Negl. (2011) 35:408–13. 10.1016/j.chiabu.2011.02.006 21652073 PMC3119733

[5] Dube SR Anda RF Felitti VJ Chapman DP Williamson DF Giles WH. Childhood abuse, household dysfunction, and the risk of attempted suicide throughout the life span: findings from the Adverse Childhood Experiences Study. JAMA. (2001) 286:3089–96. 10.1001/jama.286.24.3089 11754674

[6] Merrick MT Ports KA Ford DC Afifi TO Gershoff ET Grogan-Kaylor A. Unpacking the impact of adverse childhood experiences on adult mental health. Child Abuse Negl. (2017) 69:10–9. 10.1016/j.chiabu.2017.03.016 28419887 PMC6007802

[7] Ryff CD Singer B. Psychological well-being: Meaning, measurement, and implications for psychotherapy research. Psychother Psychosom. (1996) 65:14–23. 10.1159/000289026 8838692

[8] Pressman SD Jenkins BN Moskowitz JT. Positive affect and health: what do we know and where next should we go? Ann Rev Psychol. (2019) 70:627–50. 10.1146/annurev-psych-010418-102955 30260746

[9] Ryff CD. Psychological well-being revisited: advances in the science and practice of eudaimonia. Psychother Psychosom. (2014) 83:10–28. 10.1159/000353263 24281296 PMC4241300

[10] Lyubomirsky S King L Diener E. The benefits of frequent positive affect: does happiness lead to success? Psychol Bull. (2013) 131:803–55. 10.1037/0033-2909.131.6.803 16351326

[11] Wright TA Staw BM. Affect and favorable work outcomes: two longitudinal tests of the happy—productive worker thesis. J Organ Behav. (1999) 20:1–23. 10.1002/(SICI)1099-1379(199901)20:1<1::AID-JOB885>3.0.CO

[12] Ding Y Lin H Zhou L Yan H He N. Adverse childhood experiences and interaction with methamphetamine use frequency in the risk of methamphetamine-associated psychosis. Drug Alcohol Depend. (2014) 142:295–300. 10.1016/j.drugalcdep.2014.06.042 25064022

[13] Fu H Feng T Qin J Wang T Wu X Cai Y. Reported prevalence of childhood maltreatment among Chinese college students: a systematic review and meta-analysis. PLoS ONE. (2018) 13:e0205808. 10.1371/journal.pone.0205808 30321243 PMC6188789

[14] Xiao Q Dong M Yao J Li W Ye D. Parental alcoholism, adverse childhood experiences, and later risk of personal alcohol abuse among Chinese medical students. Biomed Environ Sci. (2008) 21:411–9. 10.1016/S0895-3988(08)60062-8 19133615

[15] Zhang L Mersky JP Topitzes J. Adverse childhood experiences and psychological well-being in a rural sample of Chinese young adults. Child Abuse Negl. (2020) 108:104658. 10.1016/j.chiabu.2020.104658 32799016

[16] Chang X Jiang X Mkandarwire T Shen M. Associations between adverse childhood experiences and health outcomes in adults aged 18-59 years. PLoS ONE. (2019) 14:e0211850. 10.1371/journal.pone.0211850 30730980 PMC6366931

[17] Cheung S Huang CC Zhang C. Passion and persistence: investigating the relationship between adverse childhood experiences and grit in college students in China. Front Psychol. (2021) 12:642956. 10.3389/fpsyg.2021.642956 33692733 PMC7937715

[18] Cui N Cao F Li Y Long Z Li J Dong F. Yixue xinsheng de ertongqi buliang jingli de leiji xiaoying yu gongji xingwei [The accumulative effect of adverse childhood experiences and aggressive behaviors among medical college students] Chin Mental Health J. (2013) 27:213–4. 10.3969/j.issn.1000-6729.2013.03.011

[19] Fung HW Ross CA Yu CKC Lau EKL. Adverse childhood experiences and dissociation among Hong Kong mental health service users. J Trauma Dissoc. (2019) 20:457–70. 10.1080/15299732.2019.1597808 30945625

[20] Anda R Tietjen G Schulman E Felitti V Croft J. Adverse childhood experiences and frequent headaches in adults. Headache J Head Face Pain. (2010) 50:1473–81. 10.1111/j.1526-4610.2010.01756.x 20958295

[21] Chapman DP Whitfield CL Felitti VJ Dube SR Edwards VJ Anda RF. Adverse childhood experiences and the risk of depressive disorders in adulthood. J Affect Disord. (2004) 82:217–25. 10.1016/j.jad.2003.12.013 15488250

[22] Felitti VJ Anda RF Nordenberg D Williamson DF Spitz AM Edwards V. Relationship of childhood abuse and household dysfunction to many of the leading causes of death in adults: the Adverse Childhood Experiences (ACE) study. Am J Prev Med. (1998) 14:245–58. 10.1016/S0749-3797(98)00017-8 9635069

[23] Huang CC Tan Y Cheung SP Hu H. Adverse childhood experiences and psychological well-being in Chinese college students: mediation effect of mindfulness. Int J Environ Res Public Health. (2021) 18:1636. 10.3390/ijerph18041636 33572110 PMC7915366

[24] Wang YR Sun JW Lin PZ Zhang HH Mu GX Cao FL. Suicidality among young adults: Unique and cumulative roles of 14 different adverse childhood experiences. Child Abuse Negl. (2019) 98:104183. 10.1016/j.chiabu.2019.104183 31521907

[25] Liu M Mejia-Lancheros C Lachaud J Latimer E Aubry T Somers J. Overall and gender-specific associations between dimensions of adverse childhood experiences and mental health outcomes among homeless adults: associations Générales et Sexospécifiques Entre les Dimensions des Expériences Défavorables de L'enfance et les Résultats de Santé Mentale Chez les Adultes Sans Abri. Can J Psychiatry. (2021). 10.1177/0706743721989158. [Epub ahead of print]. 33502253 PMC8573704

[26] Loudermilk E Loudermilk K Obenauer J Quinn MA. Impact of adverse childhood experiences (ACEs) on adult alcohol consumption behaviors. Child Abuse Negl. (2018) 86:368–74. 10.1016/j.chiabu.2018.08.006 30241703

[27] Westermair AL Stoll AM Greggersen W Kahl KG Hüppe M Schweiger U. All unhappy childhoods are unhappy in their own way—differential impact of dimensions of adverse childhood experiences on adult mental health and health behavior. Front Psychiatry. (2018) 9:198. 10.3389/fpsyt.2018.00198 29875707 PMC5974933

[28] He X Hang Ng K. In the name of harmony: the erasure of domestic violence in China's judicial mediation. Int J Law Policy Family. (2013) 27:97–115. 10.1093/lawfam/ebs014

[29] Yick AG. Predictors of physical spousal/intimate violence in Chinese American families. J Fam Violence. (2000) 15:249–67. 10.1023/A:1007501518668

[30] Bajaj B Pande N. Mediating role of resilience in the impact of mindfulness on life satisfaction and affect as indices of subjective well-being. Pers Individ Dif. (2016) 93:63–7. 10.1016/j.paid.2015.09.005

[31] Rutter M. Implications of resilience concepts for scientific understanding. Ann N Y Acad Sci. (2006) 1094:1–12. 10.1196/annals.1376.002 17347337

[32] Wagnild GM Young HM. Development and psychometric evaluation of the resilience scale. J Nurs Meas. (1993) 1:165–78. 7850498

[33] Kelifa MO Yang Y Herbert C He Q Wang P. Psychological resilience and current stressful events as potential mediators between adverse childhood experiences and depression among college students in Eritrea. Child Abuse Negl. (2020) 106:104480. 10.1016/j.chiabu.2020.104480 32470689

[34] Southwick SM Bonanno GA Masten AS Panter-Brick C Yehuda R. Resilience definitions, theory, and challenges: interdisciplinary perspectives. Eur J Psychotraumatol. (2014) 5:25338. 10.3402/ejpt.v5.25338 25317257 PMC4185134

[35] Luthar SS Cicchetti D Becker B. The construct of resilience: A critical evaluation and guidelines for future work. Child Dev. (2000) 71:543–62. 10.1111/1467-8624.00164 10953923 PMC1885202

[36] Ungar M. Resilience across cultures. Br J Soc Work. (2008) 38:218–35. 10.1093/bjsw/bcl343

[37] Herrman H Stewart DE Diaz-Granados N Berger EL Jackson B Yuen T. What is resilience? Can J Psychiatry. (2011) 56, 258–65. 10.1177/070674371105600504 21586191

[38] Herman JL. Trauma and Recovery. New York, NY: Basic Books (1992).

[39] Abiola T Udofia O. Psychometric assessment of the Wagnild and Young's resilience scale in Kano, Nigeria. BMC Res Notes. (2011) 4:1–5. 10.1186/1756-0500-4-509 22112503 PMC3261834

[40] Burns RA Anstey KJ Windsor TD. Subjective well-being mediates the effects of resilience and mastery on depression and anxiety in a large community sample of young and middle-aged adults. Austral New Zeal J Psychiatry. (2011) 45:240–8. 10.3109/00048674.2010.529604 21070186

[41] Klibert J Lamis DA Collins W Smalley KB Warren JC Yancey CT. Resilience mediates the relations between perfectionism and college student distress. J Counsel Dev. (2014) 92:75–82. 10.1002/j.1556-6676.2014.00132.x

[42] Cohn MA Fredrickson BL Brown SL Mikels JA Conway AM. Happiness unpacked: positive emotions increase life satisfaction by building resilience. Emotion. (2009) 9:361–8. 10.1037/a0015952 19485613 PMC3126102

[43] Fredrickson BL Tugade MM Waugh CE Larkin GR. What good are positive emotions in crisis? A prospective study of resilience and emotions following the terrorist attacks on the United States on September 11th, 2001. J Pers Soc Psychol. (2003) 84:365–76. 10.1037//0022-3514.84.2.365 12585810 PMC2755263

[44] Burns RA Anstey KJ. The Connor–Davidson resilience scale (CD-RISC): testing the invariance of a uni-dimensional resilience measure that is independent of positive and negative affect. Pers Individ Dif. (2010) 48:527–31. 10.1016/j.paid.2009.11.026

[45] Simpson G Jones K. How important is resilience among family members supporting relatives with traumatic brain injury or spinal cord injury? Clin Rehabilit. (2013) 27:367–77. 10.1177/0269215512457961 23012693

[46] Hu T Zhang D Wang J. A meta-analysis of the trait resilience and mental health. Pers Individ Dif. (2015) 76:18–27. 10.1016/j.paid.2014.11.039

[47] Tranter H Brooks M Khan R. Emotional resilience and event centrality mediate posttraumatic growth following adverse childhood experiences. Psychol Trauma Theor Res Pract Policy. (2021) 13:165–73. 10.1037/tra0000953 32881570

[48] Finkelhor D Shattuck A Turner HA Hamby SL. The lifetime prevalence of child sexual abuse and sexual assault assessed in late adolescence. J Adolesc Health. (2014) 55:329–33. 10.1016/j.jadohealth.2013.12.026 24582321

[49] Cavanaugh CE Petras H Martins SS. Gender-specific profiles of adverse childhood experiences, past year mental and substance use disorders, and their associations among a national sample of adults in the United States. Soc Psychiatry Psychiatr Epidemiol. (2015) 50:1257–66. 10.1007/s00127-015-1024-3 25701134 PMC4521980

[50] Haatainen KM Tanskanen A Kylmä J Honkalampi K Koivumaa-Honkanen H Hintikka J. Gender differences in the association of adult hopelessness with adverse childhood experiences. Soc Psychiatry Psychiatr Epidemiol. (2003) 38:12–7. 10.1007/s00127-003-0598-3 12563554

[51] Centers for Disease Control and Prevention. Preventing Adverse Childhood Experiences. (2020). Available online at: https://www.cdc.gov/violenceprevention/aces/fastfact.html?CDC_AA_refVal=https%3A%2F%2Ffastfact.html (accessed May 19, 2021).

[52] Wagnild G. The resilience scale user's guide: for the U.S. english version of the resilience ScaleTM and the 14-Item Resilience ScaleTM (RS-14TM). Worden, MT: The Resilience Center (2016).

[53] Aiena BJ Baczwaski BJ Schulenberg SE Buchanan EM. Measuring resilience with the RS−14: a tale of two samples. J Pers Assess. (2015) 97:291–300. 10.1080/00223891.2014.951445 25257682

[54] Damásio BF Borsa JC da Silva JP. 14-item resilience scale (RS-14): psychometric properties of the Brazilian version. J Nurs Meas. (2011) 19:131–45. 10.1891/1061-3749.19.3.131 22372090

[55] Pritzker S Minter A. Measuring adolescent resilience: an examination of the cross-ethnic validity of the RS-14. Child Youth Serv Rev. (2014) 44:328–33. 10.1016/j.childyouth.2014.06.022

[56] Shi M Wang X Bian Y Wang L. The mediating role of resilience in the relationship between stress and life satisfaction among Chinese medical students: a cross-sectional study. BMC Med Educ. (2015) 15:16. 10.1186/s12909-015-0297-2 25890167 PMC4332721

[57] Tian J Hong JS. Validation of the Chinese version of the resilience scale and its cutoff score for detecting low resilience in Chinese cancer patients. Support Care Cancer. (2013) 21:1497–502. 10.1007/s00520-012-1699-x 23274927

[58] Lei M Li C Xiao X Qiu J Dai Y Zhang Q. Evaluation of the psychometric properties of the Chinese version of the Resilience Scale in Wenchuan earthquake survivors. Compr Psychiatry. (2012) 53:616–22. 10.1016/j.comppsych.2011.08.007 22001021

[59] Shi M Liu L Wang ZY Wang L. Prevalence of depressive symptoms and its correlations with positive psychological variables among Chinese medical students: an exploratory cross-sectional study. BMC Psychiatry. (2016) 16:3. 10.1186/s12888-016-0710-3 26754773 PMC4707780

[60] Arbuckle JL. IBM SPSS Amos 23 user's guide. Chicago, IL: SPSS (2014).

[61] Grewal R Cote JA Baumgartner H. Multicollinearity and measurement error in structural equation models: implications for theory testing. Mark. Sci. (2004) 23:519–29. 10.1287/mksc.1040.0070

[62] Schilling EA Aseltine RH Gore S. Adverse childhood experiences and mental health in young adults: a longitudinal survey. BMC Public Health. (2007) 7:30. 10.1186/1471-2458-7-30 17343754 PMC1832182

[63] Fang X Fry DA Ji K Finkelhor D Chen J Lannen P. The burden of child maltreatment in China: a systematic review. Bull World Health Organ. (2015) 93:176–85C. 10.2471/BLT.14.140970 25838613 PMC4371492

[64] Woods-Jaeger BA Cho B Sexton CC Slagel L Goggin K. Promoting resilience: Breaking the intergenerational cycle of adverse childhood experiences. Health Educ. Behav. (2018) 45:772–80. 10.1177/1090198117752785 29433342

[65] DuMont K Mitchell-Herzfeld S Greene R Lee E Lowenfels A Rodriguez M. Healthy Families New York (HFNY) randomized trial: effects on early child abuse and neglect. Child Abuse Negl. (2008) 32:295–315. 10.1016/j.chiabu.2007.07.007 18377991

[66] Eckenrode J Ganzel B Henderson CR Jr Smith E Olds DL Powers J. Preventing child abuse and neglect with a program of nurse home visitation: the limiting effects of domestic violence. JAMA. (2000) 284:1385–91. 10.1001/jama.284.11.1385 10989400

[67] Larkin H Shields JJ Anda RF. The health and social consequences of adverse childhood experiences (ACE) across the lifespan: an introduction to prevention and intervention in the community. J Prevent Intervent Commun. (2012) 40:263–70. 10.1080/10852352.2012.707439 22970779

[68] Chandler GE Roberts SJ Chiodo L. Resilience intervention for young adults with adverse childhood experiences. J Am Psychiatr Nurses Assoc. (2015) 21:406–16. 10.1177/1078390315620609 26711904

[69] Chmitorz A Kunzler A Helmreich I Tüscher O Kalisch R Kubiak T. Intervention studies to foster resilience–a systematic review and proposal for a resilience framework in future intervention studies. Clin Psychol Rev. (2018) 59:78–100. 10.1016/j.cpr.2017.11.002 29167029

[70] Steinhardt M Dolbier C. Evaluation of a resilience intervention to enhance coping strategies and protective factors and decrease symptomatology. J Am Coll Health. (2008) 56:445–53. 10.3200/JACH.56.44.445-454 18316290

[71] Ji K Finkelhor D Dunne M. Child sexual abuse in China: a meta-analysis of 27 studies. Child Abuse Negl. (2013) 37:613–22. 10.1016/j.chiabu.2013.03.008 23643201

[72] Lin D Li X Fan X Fang X. Child sexual abuse and its relationship with health risk behaviors among rural children and adolescents in Hunan, China. Child Abuse Negl. (2011) 35:680–7. 10.1016/j.chiabu.2011.05.006 21907409

[73] Song Y Zhang J Zhang X. Cultural or institutional? Contextual effects on domestic violence against women in rural China. J Family Violence. (2020). 10.1007/s10896-020-00198-6. [Epub ahead of print].

[74] Fisher RJ. Social desirability bias and the validity of indirect questioning. J Consum Res. (1993) 20:303–15. 10.1086/209351

[75] Schwarz N. Self-reports: how the questions shape the answers. Am Psychol. (1999) 54:93–105. 10.1037/0003-066X.54.2.93

